# Macrophage Activation Syndrome Upon Initiation of Adalimumab in a Patient With Longstanding Rheumatoid Arthritis

**DOI:** 10.7759/cureus.12825

**Published:** 2021-01-20

**Authors:** Celestin Chicos, Milana Zirkiyeva, Sabiha Bandagi, Adriana Abrudescu

**Affiliations:** 1 Medicine, Icahn School of Medicine at Mount Sinai, Queens Hospital Center, New York, USA; 2 Rheumatology, Icahn School of Medicine at Mount Sinai, Queens Hospital Center, New York, USA

**Keywords:** rheumatoid arthritis, biologics, macrophage activation syndrome, hemophagocytic lymphohistiocytosis, adalimumab

## Abstract

Macrophage activation syndrome (MAS) is a subset of hemophagocytic lymphohistiocytosis (HLH) described in patients with rheumatological disorders. Some triggers of MAS and HLH include infection, malignancy, rheumatological disease, HIV, and rarely medications such as immunosuppressants. In recent medical literature, biologic agents are increasingly recognized as a potential trigger, but the mechanism behind this remains poorly understood. We describe the case of a patient who developed MAS after initiating adalimumab and propose a potential pathophysiological link between biologics and this syndrome.

## Introduction

In hemophagocytic lymphohistiocytosis (HLH), excessive immune activation of macrophages and impaired cytotoxic ability of natural killer (NK) cells (which are responsible for macrophage elimination and immune homeostasis) drive the phagocytosis of blood cell precursors causing severe and refractory pancytopenia. Activated macrophages proliferate in tissues and induce a cytokine storm leading to multiorgan failure which is responsible for the high mortality in this disease. Although more frequently observed in the pediatric population, the syndrome has been increasingly diagnosed in adults of all ages. Several triggers of macrophage activation syndrome (MAS) and HLH have been described in the literature, either infectious through their immune-activating effect [Epstein-Barr virus (EBV), cytomegalovirus (CMV), bacterial or fungal diseases] or conditions that lead to immunodeficiency, such as malignancy, rheumatological disorders, HIV, and rarely the use of immunosuppressants - amongst them, biologic agents such as adalimumab [1, 2], etanercept [3], canakinumab [4] and the immune checkpoint inhibitors nivolumab and ipilimumab [5]. We are reporting the case of a patient with longstanding rheumatoid arthritis (RA) who developed MAS after initiation of adalimumab.

## Case presentation

A 74-year-old female with hypertension, diabetes mellitus, chronic kidney disease and recently diagnosed rheumatoid arthritis who immigrated to the United States one year prior from Bangladesh presented with malaise, nausea, diarrhea, chills and decreased appetite. For the past six months since being diagnosed with rheumatoid arthritis, she was treated with prednisone and methotrexate, which she could not tolerate due to gastrointestinal side effects. Methotrexate was stopped and switched to leflunomide; however, the patient then developed transaminitis and thrombocytopenia to 60s. In either case, both disease-modifying anti-rheumatic drugs (DMARDs) failed to induce remission in her disease and were stopped due to intolerance. Adalimumab was started along with prednisone leading to successful control in her disease.

Two months after adalimumab was initiated, the patient presented to the emergency department as febrile to 39.7 C and was admitted to the hospital with fever of unknown origin. No signs of arthritic flare were present on our physical examination. Laboratory studies revealed leukopenia (3.39) with an absolute neutrophil count of 1460 and 22% bands, thrombocytopenia (54), anemia [hemoglobin (Hb) 9.8, hematocrit (HCT) 29.6] with a positive Coombs test, high inflammatory markers [C-reactive protein (CRP) 60.7], low fibrinogen (97), markedly elevated ferritin (87.718), transaminitis (AST 201, ALT 61 with negative HAV, HBV, undetectable HCV RNA quantitative assay), high IL-6 (63.7), lactate dehydrogenase (LDH) (above 900 IU/L) and d-dimer (34.089). She was started on broad-spectrum antibiotics and intravenous methylprednisolone, but extensive infectious work-up (including blood and urine cultures, EBV and Quantiferon-TB) failed to reveal a particular source. COVID-19 was twice negative, and a negative chest computed tomography angiography (CTA) and lower extremity Doppler made venous thromboembolism unlikely. Occult malignancy was ruled out with negative computer tomography of the chest, abdomen and pelvis. Rheumatological work-up included a positive antinuclear antibody (ANA 1:320, speckled pattern) with negative anti-double stranded DNA, negative anti-Smith antibodies and normal complement levels (C3 complement 124, C4 complement 58). Although anti-histone antibodies (6.3, ref. range 0.0-0.9 units) were positive, the patient continued to be febrile and pancytopenic despite steroid therapy, therefore we deemed drug-induced lupus less likely given normal complement levels. In addition, the patient displayed nephrotic-range proteinuria (4.3 g/L) with worsening of her baseline kidney function (creatinine 2.5, from baseline 2.0) and kidney biopsy showed glomerulosclerosis without signs of lupus nephritis or amyloidosis, and stained negatively under Congo red. Anti-neutrophil cytoplasmic antibodies (c-ANCA and p-ANCA) were negative. Of note, patient’s fasting triglycerides were elevated at 217 mg/dL, and soluble CD25 returned high at 4.363 (ref. range 175-858 pg/mL). Given refractory laboratory abnormalities meeting five out of eight diagnostic criteria for MAS/HLH [6] - hyperferritinemia, elevated fasting triglycerides, pancytopenia, hypofibrinogenemia and high soluble CD25 - bone marrow biopsy was performed. Pathology confirmed hemophagocytosis with focally prominent histiocytes, many containing cellular debris and phagocytosed red blood cells. A diagnosis of macrophage activation syndrome was made and therapy was initiated with dexamethasone and etoposide [7].

## Discussion

Macrophage activation syndrome (MAS) is a life-threatening disease that can be triggered by infection, malignancy, autoimmune disease and rarely medications such as biologic agents. Our particular case had longstanding, undiagnosed and untreated rheumatoid arthritis and treatment was started six months prior, at the time of RA diagnosis but late in the course of her disease. After initial DMARDs failed to control the symptoms, adalimumab was added to prednisone for the two months leading to presentation, which led to remission of her disease. Due to the temporality between initiation of adalimumab and MAS in our particular case, we favor the biologic agent as a possible trigger in the development of the syndrome, as opposed to reactive macrophage activation secondary to rheumatoid arthritis, which was controlled for months prior. While a definitive link between biologics and MAS has not been formally established, several cases in the literature report macrophage activation syndrome after initiation of etanercept, canakinumab, infliximab [8] and adalimumab. The development of MAS in a similar temporal relationship to adalimumab and without a detectable infectious trigger was also described by Baker et al. in an adult patient with axial spondyloarthritis [9]. The pathophysiologic mechanism behind this syndrome is not well understood, but it appears to involve modulation of toll-like receptors (TLRs) by biologic agents. TLRs are innate pattern recognition receptors present on the surface of cells which sense non-specific, foreign molecular components present across microbial species and activate the cell signaling pathway to induce migration of the NF-kB factor from the cytoplasm to the nucleus, leading to transcriptional expression of immune genes responsible for secretion of cytokines such as TNF alpha, interferon or interleukins and ultimately, inflammation [10]. By inhibiting TNF alpha, De Pità et al. observed that adalimumab is also able to down-regulate the activity of TLRs which are overexpressed on the surface of keratinocytes in psoriatic lesions, thus dampening inflammation and leading to clinical improvement in this disease [11]. Whether biologics alter TLR expression and affect the cell signaling pathways in a way that, on a background of genetic predisposition, leads to the dysregulated immune state seen in MAS remains to be proven, but the emergence of similar cases in the recent medical literature warrants further study.

## Conclusions

Adalimumab is known for an increased risk of lymphoproliferative disorders, and the class of biologic agents is emerging as a potential trigger for macrophage activation syndrome. Although it remains a potentially fatal disease, heightened physician awareness in recent years should lead to more rapid diagnosis and improved survival of these patients. Through our case we aim to illustrate that MAS in adult patients with rheumatological disorders might not be as rare as we once thought, and persistent laboratory abnormalities without an obvious source of infection or malignancy should prompt searching for this hemophagocytic syndrome, especially in patients that were recently started on biologics.
